# Assessment of commercially available artificial intelligence software for differentiating hemorrhage from contrast on head CT following thrombolysis for ischemic stroke

**DOI:** 10.3389/fneur.2025.1458142

**Published:** 2025-03-04

**Authors:** Ayden L. Olsen, Daniel Thomas Ginat

**Affiliations:** ^1^Pritzker School of Medicine, The University of Chicago, Chicago, IL, United States; ^2^Department of Radiology, Section of Neuroradiology, The University of Chicago, Chicago, IL, United States

**Keywords:** intracranial hemorrhage, dual energy CT, artificial intelligence, commercial AI, contrast CT

## Abstract

**Background:**

In patients who have undergone ischemic stroke therapy, retained iodine-based contrast can resemble acute intracranial hemorrhage (ICH) on standard computed tomography (CT). The purpose of this study is to determine the accuracy of commercially available artificial intelligence software for differentiating hemorrhage from contrast in such cases.

**Methods:**

A total of 45 CT scans analyzed by Aidoc software that also included dual-energy iodine subtraction maps from dual energy CT from 23 unique patients (12 male, 11 female, age range 30–99 years, mean age 67.6 years, standard deviation 18.5 years) following recent ischemic stroke therapy were retrospectively reviewed for the presence of hemorrhage versus retained contrast material.

**Results:**

The sensitivity and specificity of the model in detecting acute intracranial hemorrhage as opposed to contrast were 51.7 and 50.0%, respectively. The positive and negative predictive values were 65.2 and 36.4%, respectively.

**Conclusion:**

The current Aidoc software is not optimized for differentiating between acute hemorrhage and retained contrast on CT. This justifies the development of a more robust artificial intelligence model trained to differentiate between ICH and iodine contrast based on both DECT and standard CT images.

## Introduction

Acute intracranial hemorrhage (ICH) after intravenous thrombolytic therapy for ischemic stroke is rare but potentially life-threatening depending on the size and location of the bleed. Rapid identification and management are essential to achieving positive patient outcomes (1). Following ischemic stroke, patients may receive acute interventions such as intravenous tissue plasminogen activator (tPA) administration (e.g., alteplase or tenecteplase) or mechanical thrombectomy to restore cerebral blood flow. Diagnostic imaging prior to treatment typically involves the use of an iodine-based contrast agent to evaluate vascular occlusion, collateral flow, and perfusion status. However, blood–brain barrier (BBB) leakage can lead to retention of contrast material, which may persist in subsequent non-contrast imaging, complicating the differentiation between retained contrast and acute ICH. Post-treatment imaging, typically performed without the administration of an iodine contrast agent, aims to detecting complications such as ICH, but retained iodine contrast from pre-treatment imaging may appear as hyperdense foci on standard head CT, mimicking hemorrhage. Previous studies estimate that iodine extravasation may account for up to 84% of hyperdense foci seen on follow-up scans after non-mechanical thrombolysis for ischemic stroke (2, 3). Thus, retained contrast can present a challenge in post-treatment imaging, as it is difficult to differentiate from acute ICH.

Dual-energy computed tomography (DECT) provides a solution to this diagnostic challenge by utilizing two unique photon energy spectra to better distinguish materials with varying attenuation properties (4). With post-processing, DECT exams can be used to generate an iodine overlay map (IOM), enabling the creation of virtual non-contrast (VNC) images (5–8). These VNC images subtract densities corresponding to iodine contrast from the enhanced images, significantly aiding in the differentiation between retained iodine contrast and acute ICH. Because of its ability to generate IOMs and VNC images, DECT achieves nearly 100% accuracy in distinguishing between retained iodine contrast and hemorrhage in scenarios of post-ischemic stroke therapy (9). While VNC images generated by DECT may slightly differ in the exact attenuation values as compared to true non-contrast CT (NCCT), multiple studies have shown that these differences are relatively minor and that VNC images can be used reliably in diagnostic scenarios (10–12).

With the increasing use of artificial intelligence (AI) as a useful tool in the field of radiology over the past several years, many triage AI models have been developed and implemented in various hospitals across the world. While some of these AI models perform at very high accuracy, others are underdeveloped or not trained to a level at which they can be consistently reliable in clinical situations. In the setting of post-stroke therapy CT imaging, an AI model able to differentiate between retained contrast and acute hemorrhage would enable the flagging of cases with suspected ICH, allowing for more rapid identification and intervention, leading to better patient outcomes.

Recently, Aidoc (Tel Aviv, Israel) has created an artificial intelligence (AI) model to detect acute ICH on standard CT images, flagging images with suspected hemorrhage for further review by a trained radiologist. Despite the promisingly high performance reported (13), this model does not appear to be optimized for detecting the difference between ICH and retained contrast material on conventional CT exams. We have previously shown that the implementation of an automatic flagging system decreases scan view delay time and expedited diagnosis of urgent conditions (14). Because of the critical need to quickly identify and assess potential cases of ICH, it is important to assess the ability of the Aidoc model to accurately distinguish between ICH and retained iodine contrast, and, if it cannot, to develop a novel model that can do so with more generalizable accuracy.

In this study, we use the Aidoc model to predict contrast versus hemorrhage for 45 CT exams showing hyperdensities confirmed to be either retained contrast or acute hemorrhage. Through this analysis, we show that performance of current AI models in distinguishing between retained contrast and acute ICH on post-stroke therapy CT scans is insufficient, and a more robust model needs to be trained and validated.

## Materials and methods

### Inclusion criteria

**Figure 1:**
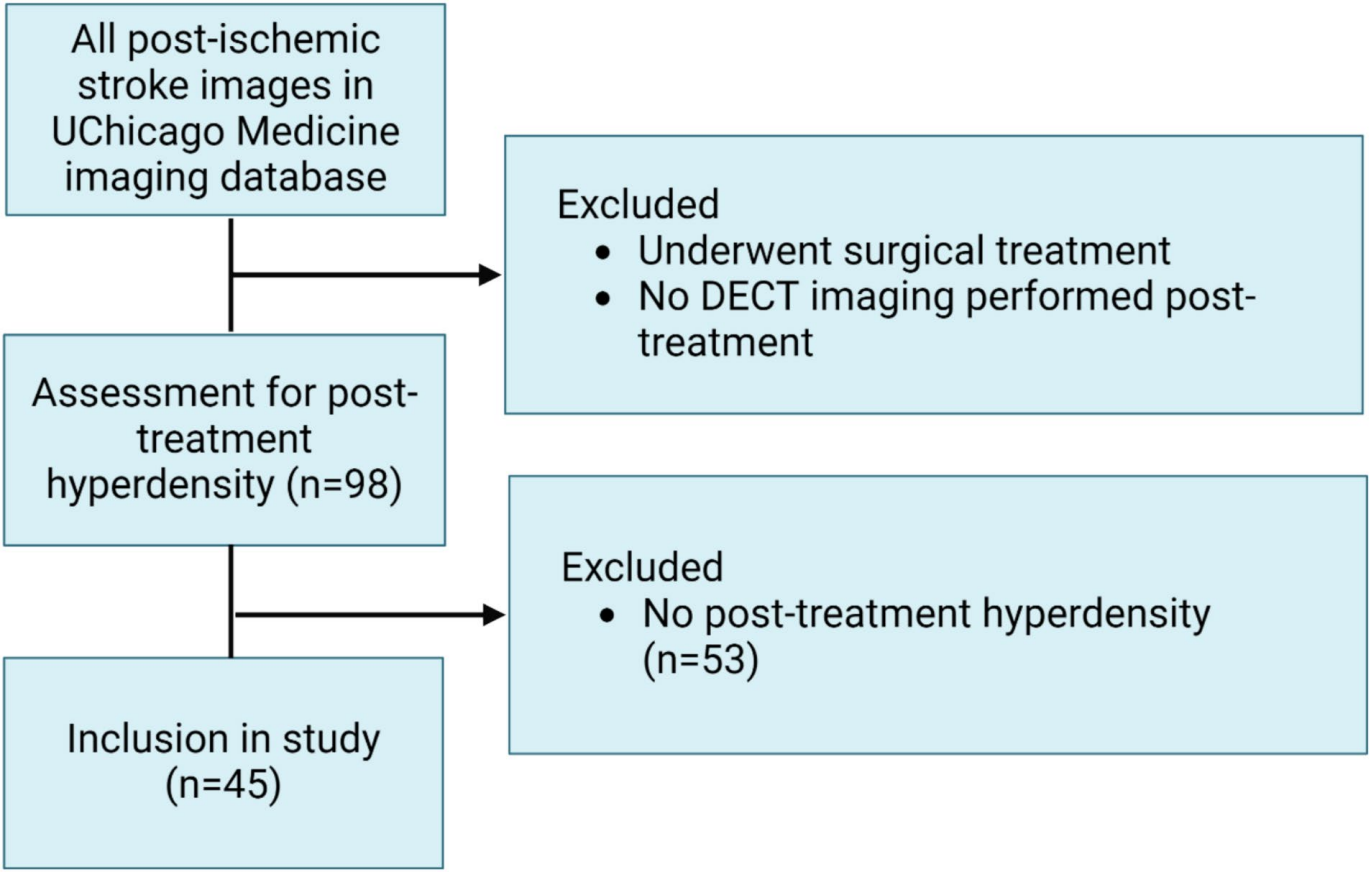
Inclusion criteria for analyzed images (Created using BioRender).

This study retrospectively analyzes CT and DECT exams of adult patients (age 18+) admitted to the University of Chicago Medical Center between January 2014 and June 2024 in the setting of acute ischemic stroke. After NCCT confirmation of an ischemic lesion, patients included in the study underwent acute tenecteplase therapy and subsequent follow-up DECT imaging at 24–48 h post-treatment to monitor for the presence of acute intracranial hemorrhage (Figure 1). Several included patients underwent multiple follow-up imaging studies (Table 1).

**Table 1:** Patients receiving multiple follow-up imaging studies.

# follow-up imaging studies	# patients	# image sets generated
1	12	12
2	5	10
3	3	9
4	1	4
5	2	10
Total	23	45

A search of the imaging database identified 98 CT image sets from 49 unique patients, some of whom underwent multiple post-treatment scans. 53 of these imaging sets (from 26 unique patients) did not show any hyperdensity and thus were excluded from analysis. The remaining 45 image sets were included in the analysis. These image sets were collected from 23 unique patients, 12 male (52%), 11 female (48%), age range 30–99 years, mean age 67.6 years, standard deviation 18.5 years.

### Imaging specifications

DECT image sets were obtained using a GE Gemstone Spectral CT in helical scan mode. Images were obtained at a tube voltage of 80/140 kVp and a tube current of 300 mA. Tube rotation time was 0.8 s and pitch was 0.52. Images were obtained at a thickness of 5 mm and with a reconstruction interval of 0.63 mm.

Standard CT image sets were obtained at a tube voltage of 140 kVp and a tube current of 405 mA. Pitch was 0.52. Images were obtained at a thickness of 0.63 mm and a reconstruction field-of-view of 299 mm with a standard reconstruction filter.

### Aidoc software

After anonymization, conventional CT image sets were fed into an FDA-approved convolutional neural network algorithm developed by Aidoc aimed at identifying ICH via slice-by-slice analysis. In prior studies, this model is reported to detect ICH with a specificity of 99%, a sensitivity of 95%, and an overall accuracy of 98% (13).

### Analysis

Both standard CT and DECT image sets for patients meeting the inclusion criteria were obtained from the imaging database. Under the guidance of an experienced neuroradiologist, DECT image sets (including an iodine suppression map) from each case were used to confirm each hyperdensity as acute ICH, retained iodine contrast, or both. The neuroradiologist’s assessment constituted the ground truth for this study. A confusion matrix was then constructed based on the predictions of the Aidoc software and the ground truth obtained from the neuroradiologist’s interpretation of the DECT image sets. Specificity, sensitivity, positive predictive value, and negative predictive value were then calculated for the Aidoc software.

### IRB statement

This study was approved by the Institutional Review Board at the University of Chicago. No patient identifiers were recorded, and all images were fully anonymized before being fed into the Aidoc software. As this study falls under the category of quality improvement research, the need for patient consent was waived.

## Results

**Figure 2:**
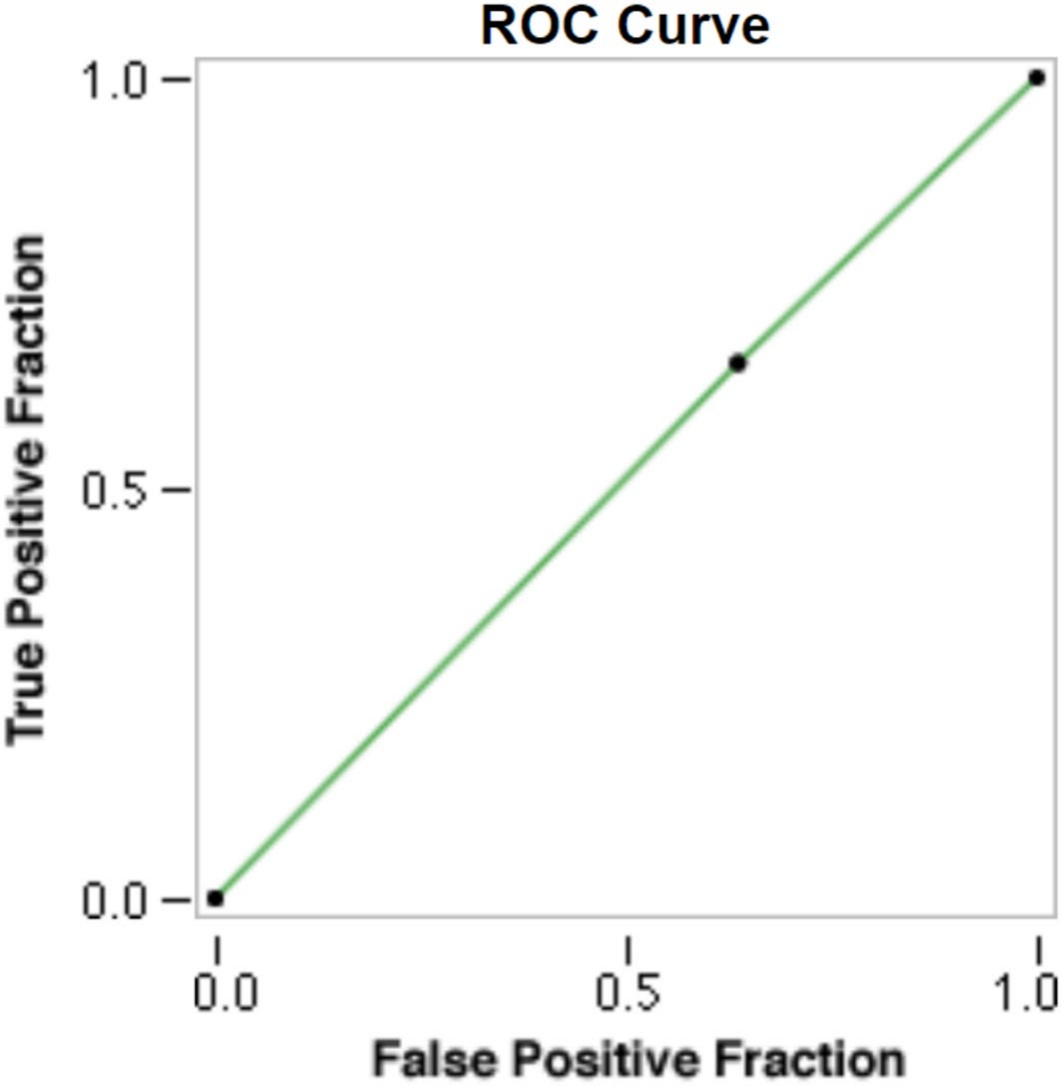
ROC curve created from the Aidoc model classifications with an AUC of 0.51.

A total of 45 image sets from 23 unique patients were fed into the Aidoc software. After receiving an image set, the software determined if the hyperdensity on the image likely represented ICH or retained iodine contrast. These results were then compared with the diagnosis made from the DECT scan. Of the 45 images screened by the software, 29 (64%) were flagged as positive for ICH, and 16 (36%) were flagged as negative. Among the 29 images flagged by the software as positive for ICH, the true positive rate was 52% (15/29), and the false positive rate was 48% (14/29). Of the 16 images screened as negative for ICH, the true negative rate was 50% (8/16) and the false negative rate was 50% (8/16) (Table 2). The sensitivity, specificity, positive predictive value, and negative predictive value were calculated as 51.7, 50.0, 65.2, and 36.4%, respectively, with an overall accuracy of 51.1% (Table 3). A receiver operating characteristic (ROC) curve was generated using the Aidoc model classification and demonstrated an area under the curve (AUC) of 0.51 (Figure 2).

**Table 2:** Confusion matrix.

	Ground truth positive (+)	Ground truth negative (−)	Total
Aidoc positive (+)	15	8	**23**
Aidoc negative (−)	14	8	**22**
Total	29	16	45

**Table 3:** Statistical calculations.

Parameter	Value	95% CI
Sensitivity	51.7%	32.5–70.6%
Specificity	50.0%	24.7–75.4%
Positive predictive value	65.2%	50.6–77.4%
Negative predictive value	36.4%	23.6–51.5%
Overall accuracy	51.1%	35.8–66.3%

## Discussion

Despite the ability of the Aidoc model to detect ICH on NCCT images as reported in the original study (13), our results show that the Aidoc deep learning model is not optimized for detecting acute ICH in the presence of possible retained iodine contrast. In the presence of contrast, the Aidoc software was only able to correctly flag 65.2% of ICH-positive cases, misjudging the remaining 34.8% as retained contrast.

Accurate differentiation between retained contrast and ICH is critical for timely and effective clinical management. A model capable of reliably distinguishing between the two could significantly reduce diagnostic errors, streamline clinical workflow, and enhance patient outcomes. Cases in which retained contrast is misclassified as ICH may lead to unnecessary interventions, delayed care for actual ICH cases, and increased costs. Conversely, false negatives, where true ICH is misclassified as contrast, could cause delay in treatment, including failure to initiate life-saving therapies such as anticoagulation reversal or surgical intervention. Integrating a highly sensitive and specific AI model into radiological workflows could enhance diagnostic accuracy, reduce radiologist workload, and minimize human oversight. For example, a model with a specificity of 98% could eliminate up to 90% of false ICH alarms, while maintaining a sensitivity of 95% could ensure timely detection of nearly all true ICH cases. Refining AI algorithms to minimize false outputs and incorporating clinician feedback into iterative development are essential steps to achieving optimal clinical impact.

**Figure 3:**
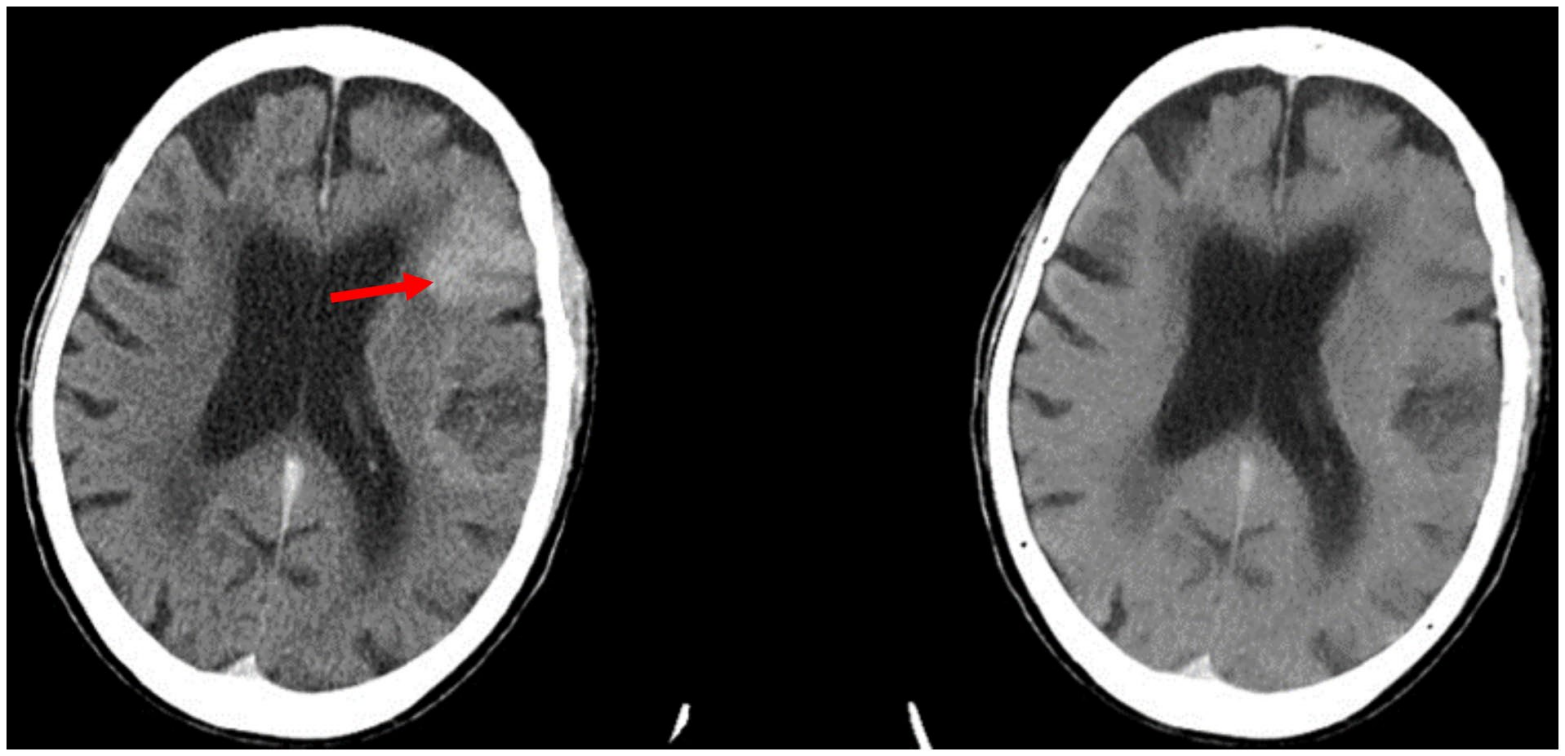
Example of a false positive reported by the Aidoc algorithm: the retained contrast in the left frontal lobe (red arrow) seen on contrast CT (left) was flagged for ICH. The iodine suppression image (right) confirms that there is no hemorrhage.

**Figure 4:**
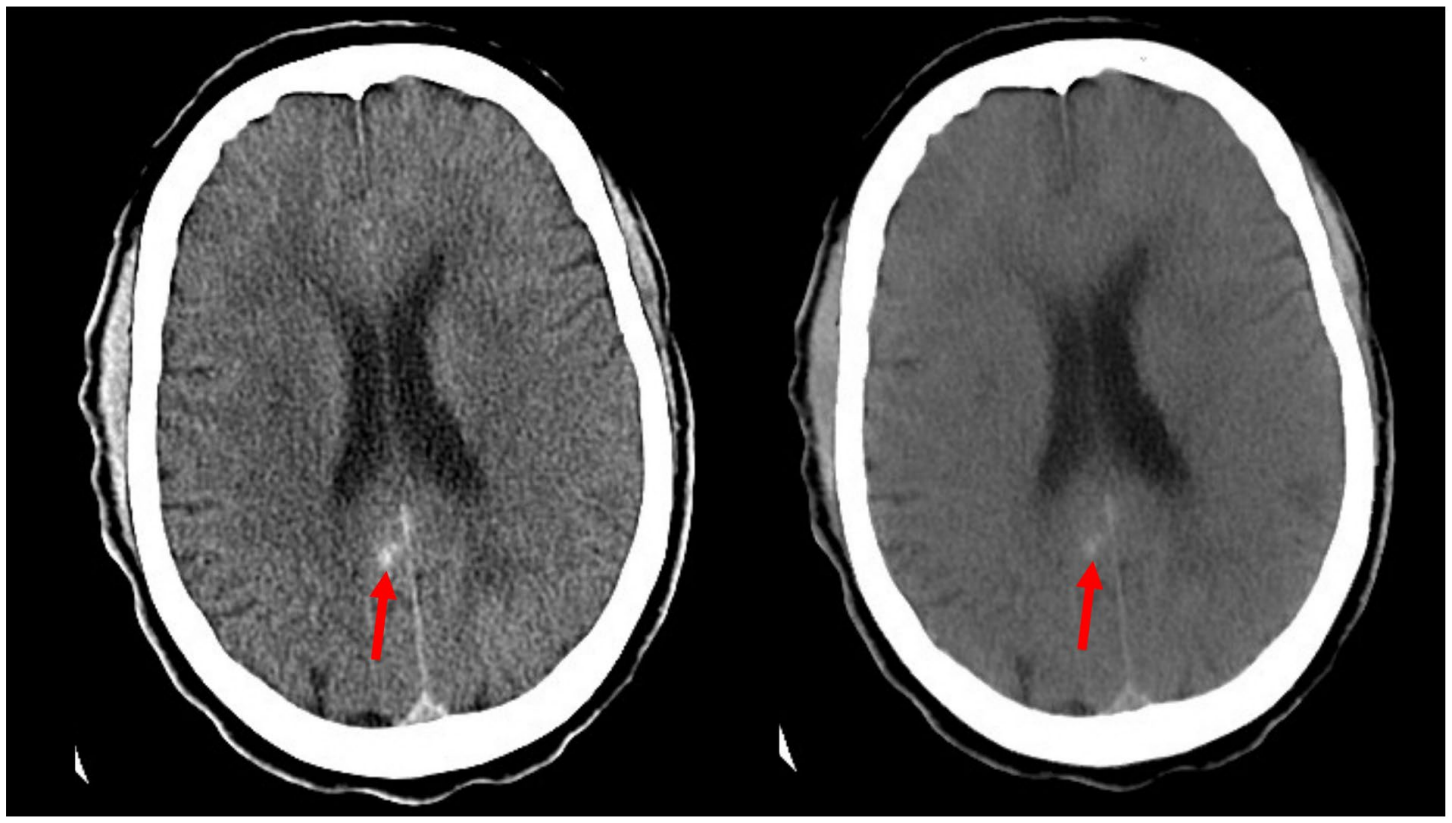
Example of a false negative reported by the Aidoc algorithm: a small area of hemorrhage along the posterior cingulate gyrus (red arrows) as seen in the contrast CT (left) is confirmed by persistence in the iodine suppression image (right).

Analysis of the image sets used in this study showed that ICH flagging seemed to follow an attenuation-based pattern. Acute ICH displays strong hyperattenuation on contrast CT, and in many false positive cases, the retained contrast was found to be particularly hyperattenuating (Figure 3). Conversely, cases of retained contrast that were correctly judged as ICH-negative tended to display contrast with weaker hyperattenuation (Figure 4). While our dataset included only three cases presenting with petechial hemorrhage, all were inappropriately judged to be ICH-negative by the Aidoc algorithm.

There are several limitations to this study. First, our study included multiple follow-up image series from several of the same patients. While these repeated scans from the same patient are not completely independent data points, we performed an additional sensitivity analysis in which only the first follow-up scan per patient was included. The sensitivity was 62.7% and the specificity was 26.7%, confirming that while there were changes to the statistical outcome, our overall claims about the accuracy of the model remained consistent. Second, the true-positive and false-negative rates may be exaggerated due to an overrepresentation of patients presenting with ICH at follow-up in comparison to patients without ICH. Additionally, the standard imaging indication for post-thrombolytic therapy is NCCT rather than contrast CT or DECT, leading to the availability of a small number of cases for analysis. To confirm that the findings of this study are generalizable, more DECT scans from post-tenecteplase patients should be fed through the algorithm, ideally from a broad range of locations. Despite the limitations of this analysis, the poor performance of the Aidoc system in distinguishing between acute hemorrhage and retained iodine contrast is not ideal. Finally, the ground truth was determined from DECT images alone instead of MRI.

The development of a more robust AI detection system is necessary to reduce the time radiologists spend reviewing critical cases. A more optimized AI model could help prioritize urgent cases, ensuring that potentially life-threatening conditions such as ICH are flagged quickly for immediate attention. This could prevent delays and reduce the risk of congestion on ‘stat’ reading lists, where time-sensitive cases are often very high-volume yet still require swift interpretation to guide clinical decisions.

## Conclusion and future directions

Our analysis shows that the Aidoc software was unable to consistently differentiate between acute ICH and retained iodine contrast on post-tPA CT images. We believe this warrants the development of a more robust AI detection system that can be deployed to flag the images of patients with potential ICH, leading to quicker treatment and more favorable outcomes.

## Data Availability

The raw data supporting the conclusions of this article will be made available by the authors, without undue reservation.

## References

[1] Yaghi S Willey JZ Cucchiara B Goldstein JN Gonzales NR Khatri P. American Heart Association stroke council; council on cardiovascular and stroke nursing; council on clinical cardiology; and council on quality of care and outcomes research. Treatment and outcome of hemorrhagic transformation after intravenous Alteplase in acute ischemic stroke: a scientific statement for healthcare professionals from the American Heart Association/American Stroke Association. Stroke. (2017) 48:e343–61. doi: 10.1161/STR.0000000000000152 29097489

[2] Liu K Jiang L Ruan J Xia W Huang H Niu G. The role of dual energy CT in evaluating hemorrhagic complications at different stages after thrombectomy. Front Neurol. (2020) 11:583411. doi: 10.3389/fneur.2020.583411, PMID: 33117268 PMC7575741

[3] Pham J Gan C Dabboucy J Stella DL Dowling R Yan B. Occult contrast retention post-thrombectomy on 24-h follow-up dual-energy CT: associations and impact on imaging analysis. Int J Stroke. (2023) 18:1228–37. doi: 10.1177/17474930231182018, PMID: 37260232

[4] Flohr TG McCollough CH Bruder H Petersilka M Gruber K Süss C. First performance evaluation of a dual-source CT (DSCT) system. Eur Radiol. (2006) 16:256–68. doi: 10.1007/s00330-005-2919-2, PMID: 16341833

[5] Ananthakrishnan L Rajiah P Ahn R Rassouli N Xi Y Soesbe TC. Spectral detector CT-derived virtual non-contrast images: comparison of attenuation values with unenhanced CT. Abdom Radiol. (2017) 42:702–9. doi: 10.1007/s00261-016-1036-9, PMID: 28084546

[6] Chilamkurthy S Ghosh R Tanamala S Biviji M Campeau NG Venugopal VK. Deep learning algorithms for detection of critical findings in head CT scans: a retrospective study. Lancet. (2018) 392:2388–96. doi: 10.1016/S0140-6736(18)31645-3, PMID: 30318264

[7] Niehoff JH Woeltjen MM Laukamp KR Borggrefe J Kroeger JR. Virtual non-contrast versus true non-contrast computed tomography: initial experiences with a photon counting scanner approved for clinical use. Diagnostics. (2021) 11:2377. doi: 10.3390/diagnostics11122377, PMID: 34943613 PMC8700090

[8] Sauter AP Muenzel D Dangelmaier J Braren R Pfeiffer F Rummeny EJ. Dual-layer spectral computed tomography: virtual non-contrast in comparison to true non-contrast images. Eur J Radiol. (2018) 104:108–14. doi: 10.1016/j.ejrad.2018.05.007, PMID: 29857855

[9] Phan CM Yoo AJ Hirsch JA Nogueira RG Gupta R. Differentiation of hemorrhage from iodinated contrast in different intracranial compartments using dual-energy head CT. AJNR Am J Neuroradiol. (2012) 33:1088–94. doi: 10.3174/ajnr.A2909, PMID: 22268092 PMC8013231

[10] Gariani J Cuvinciuc V Courvoisier D Krauss B Mendes Pereira V Sztajzel R. Diagnosis of acute ischemia using dual energy CT after mechanical thrombectomy. J Neurointerv Surg. (2016) 8:996–1000. doi: 10.1136/neurintsurg-2015-011988, PMID: 26534867

[11] Hixson HR Leiva-Salinas C Sumer S Patrie J Xin W Wintermark M. Utilizing dual energy CT to improve CT diagnosis of posterior fossa ischemia. J Neuroradiol. (2016) 43:346–52. doi: 10.1016/j.neurad.2016.04.001, PMID: 27255679

[12] Noguchi K Itoh T Naruto N Takashima S Tanaka K Kuroda S. A novel imaging technique (X-map) to identify acute ischemic lesions using noncontrast dual-energy computed tomography. J Stroke Cerebrovasc Dis. (2017) 26:34–41. doi: 10.1016/j.jstrokecerebrovasdis.2016.08.025, PMID: 27639587

[13] Ojeda P Zawaideh M Mossa-Basha M Haynor D. The utility of deep learning: evaluation of a convolutional neural network for detection of intracranial bleeds on non-contrast head computed tomography studies In: Proc. SPIE 10949, medical imaging 2019: Image processing (2019). 109493J.

[14] Ginat D. Implementation of machine learning software on the radiology worklist decreases scan view delay for the detection of intracranial hemorrhage on CT. Brain Sci. (2021) 11:832. doi: 10.3390/brainsci11070832, PMID: 34201775 PMC8301803

